# Ulinastatin Inhibits Osteoclastogenesis and Suppresses Ovariectomy-Induced Bone Loss by Downregulating uPAR

**DOI:** 10.3389/fphar.2018.01016

**Published:** 2018-09-07

**Authors:** Jun-ming Huang, Ran-yue Ren, Yuan Bao, Jia-chao Guo, Wei Xiang, Xing-zhi Jing, Jia Shi, Guo-xiang Zhang, Long Li, Yong Tian, Hao Kang, Feng-jin Guo

**Affiliations:** Department of Orthopedics, Tongji Hospital, Tongji Medical College, Huazhong University of Science and Technology, Wuhan, China

**Keywords:** ulinastatin, uPAR, osteoclast, MAPKs, NF-κB, RANKL, osteoporosis

## Abstract

Recent studies indicate that uPAR acts a crucial part in cell migration and the modulation of bone homeostasis. As a natural serine protease inhibitor, ulinastatin owns the capacity to reduce proinflammatory factors, downregulate the activation of NF-κB and mitogen-activated protein kinases (MAPKs) signaling pathways. Osteoclastogenesis has been demonstrated to be related with low-grade inflammation which involves cell migration, thus we speculate that ulinastatin may have a certain kind of impact on uPAR so as to be a potential inhibiting agent of osteoclastogenesis. In this research, we investigated the role which ulinastatin plays in RANKL-induced osteoclastogenesis both *in vivo* and *in vitro*. Ulinastatin inhibited osteoclast formation and bone resorption in a dose-dependent manner in primary bone marrow-derived macrophages (BMMs), and knockdown of uPAR could completely repress the formation of osteoclasts. At the molecular level, ulinastatin suppressed RANKL-induced activation of cathepsin K, TRAP, nuclear factor-κB (NF-κB) and MAPKs, and decreased the expression of uPAR. At the meantime, ulinastatin also decreased the expression of osteoclast marker genes, including cathepsin K, TRAP, RANK, and NFATc1. Besides, ulinastatin prevented bone loss in ovariectomized C57 mice by inhibiting the formation of osteoclasts. To sum up, this research confirmed that ulinastatin has the ability to inhibit osteoclastogenesis and prevent bone loss, and uPAR plays a crucial role in that process. Therefore, ulinastatin could be chosen as an effective alternative therapeutics for osteoclast-related diseases.

## Introduction

Bone is not changeless in human body but unceasingly undergoes bone formation and bone resorption, respectively, caused by osteoblasts and osteoclasts, namely bone remodeling (Boyle et al., 2003). This process, which is so-called bone homeostasis, is regulated by diverse factors. Osteoclasts, which are derived from bone marrow-derived macrophages/monocytes (BMMs) (Szekanecz and Koch, 2007), are multi-nucleated cells and act a pivotal part in resorbing bone tissue (Kikuta and Ishii, 2013). Two vital cytokines which participate in differentiation and function of osteoclasts are receptor activator of nuclear factor-κB (NF-κB) ligand (RANKL) and macrophage colony-stimulating factor (M-CSF). M-CSF sustains the survival and proliferation of osteoclast precursor cells by motivating ERK and PI3K/Akt (Karsenty and Wagner, 2002) and promotes RANK expression, meanwhile, RANKL binds to RANK which is expressed on osteoclast precursor cells and mature osteoclasts resulting in the accumulation and recruitment of adaptor molecules——TNF receptor-associated factors (TRAFs) peculiarly TRAF6 (Nakashima et al., 2011). The complex RANKL-RANK-TRAF6 activates NF-κB and mitogen-activated protein kinases (MAPKs) signaling pathways, nuclear factor of activated T cells cytoplasmic 1 (NFATc1) and c-Fos (Lee et al., 2001). After activation of all these transcription factors, the expression of multifarious osteoclast marker genes is stimulated, including cathepsin K, NFATc1, tartrate-resistant acid phosphatase (TRAP), c-Fos, and matrix metalloproteinase 9 (MMP-9), which enhance the process of osteoclastogenesis and ultimately lead to bone resorption (Takayanagi, 2007b; Conaway et al., 2016).

The dysregulation of osteoclast formation and activation often causes bone destructive diseases, for instance, postmenopausal osteoporosis, rheumatoid arthritis, periodontitis, and Paget’s disease (Takayanagi, 2007a; Novack and Teitelbaum, 2008). Hence, inhibiting the abnormal formation and activation of osteoclasts could be one of the most effective strategies for treating the diseases in which osteoclasts actively participate.

The urokinase plasminogen activator receptor (uPAR), composed of three cysteine-rich domains (D1, D2, and D3), is a glycosylphosphatidylinositol (GPI)-anchored protein with a molecular weight (MW) of 55 to 70 kDa. uPAR is a multifunctional receptor on cell surface and wildly detected on endothelial cells, monocytes, fibroblasts, and multifarious malignant cells (Rao et al., 2013). uPAR can specifically bind to uPA and direct the activity of the plasminogen activation system, which leads to plasmin generation and degradation of extracellular matrix. In addition to regulation of proteolysis, uPAR promotes several intercellular signaling by the interaction with transmembrane proteins such as integrins, tyrosine kinase receptors, and others to trigger changes in cellular adhesion, differentiation, proliferation, migration, and cell survival (Blasi and Carmeliet, 2002; Smith and Marshall, 2010). The upregulation of uPAR is not only obvious in many human cancers and inflammatory diseases but also significant in poor prognosis and early invasion and metastasis (Mazar, 2008). In non-small cell lung (NSCLC) and colorectal cancer (CRC), overexpressed uPAR had been demonstrated in primary tumors and serum (Montuori et al., 2003; Lomholt et al., 2009; Almasi et al., 2011). In rheumatoid arthritis, increased uPAR was also reported in synovial tissues, synovial fluid and serum and elevated uPAR markedly increase the proliferation, migration, and invasiveness of fibroblast-like synoviocytes (Liu et al., 2018). Furthermore, downregulation of uPAR can inhibits migration, invasion, proliferation of papillary thyroid carcinoma cells (Nowicki et al., 2011). In bone tissue, expression of uPAR mediates and affects differentiation of bone marrow stem cells to osteoblasts and propagation of the osteogenic process as well as formation of osteoclast, so uPAR plays a crucial role in maintaining bone homeostasis (Furlan et al., 2007; Kalbasi et al., 2014). As mentioned above, bone destructive diseases correlate with the remarkable formation of osteoclasts which is an essential factor for unbalance between bone formation and bone resorption and then leads to bone loss. Meanwhile, the plasminogen/plasmin system plays a role in bone remodeling by regulating MMPs (Zhang et al., 2003). Thus we speculate that uPAR may be a helpful therapeutic target for these diseases.

Known as a natural serine protease inhibitor secreted by human liver (Hirose et al., 1998), ulinastatin has an inhibiting effect on multiple inflammatory proteases, such as trypsin, neutrophil elastase, chymotrypsin, and plasmin (Nakahama et al., 1999; Umeadi et al., 2008), and possesses the ability to stabilize lysosomal membrane so as to suppress the release of lysosomal enzymes, eliminate free oxygen radicals, and inhibit the release of inflammatory mediators (Kato et al., 1998; Nakahama et al., 1999). The content of inflammatory proteases elevates in the present of infection and inflammation, so the protease-inhibiting capacity of ulinastatin makes it a valid anti-inflammatory drug (Pugia and Lott, 2005). As it can be seen in the previous studies, ulinastain owns the capacity to reduce proinflammatory factors (Kato et al., 1998; Nakahama et al., 1999), downregulate the activation of NF-κB and MAPKs signaling pathways (Zhang et al., 2011), thus we speculate that ulinastatin may have a certain kind of impact on uPAR so as to be a potential inhibiting agent of osteoclastogenesis. In this research, we used ovariectomized C57 mice animal model to explore the role which ulinastatin plays in postmenopausal osteoporosis, and also performed the underlying molecular mechanism.

## Results

### Ulinastatin Inhibits OVX-Induced Osteoclast Activity and Bone Loss

**FIGURE 1:**
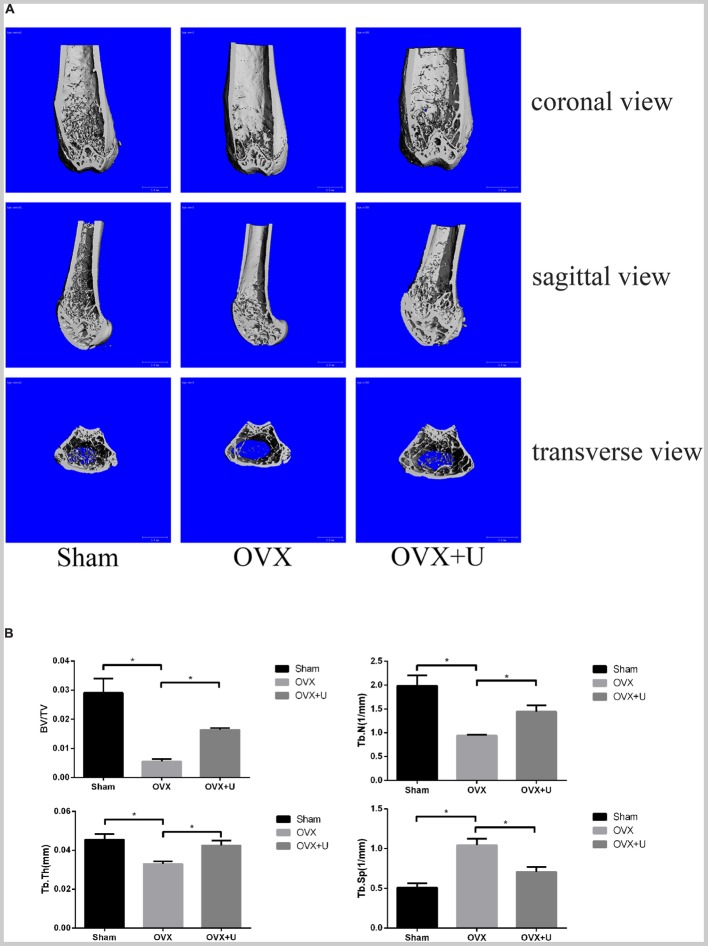
Ulinastatin inhibits bone loss induced by OVX. **(A)** μ-CT images of the trabecular bone of distal femoral metaphysis from the SHAM, OVX, and OVX + ulinastatin groups. **(B)** The trabecular structural parameters of the distal femur: trabecular bone volume/tissue volume (BV/TV), trabecular number (Tb.N), trabecular thickness (Tb.Th), and trabecular separation (Tb.Sp). Data are presented as means ±*SD*. *n* = 10 and ^∗^*P* < 0.05.

**FIGURE 2:**
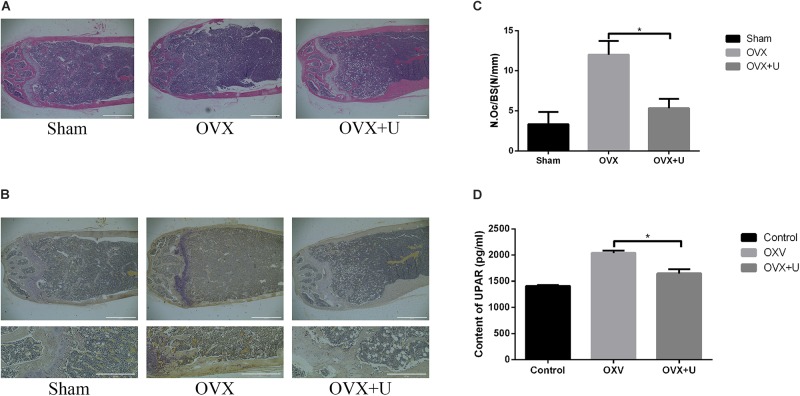
Ulinastatin inhibits the formation of osteoclasts and bone loss in OVX mice, meanwhile decreases the serum level of uPAR. **(A)** Distal femurs were embedded with paraffin and sliced up, then H&E and TRAP staining were performed. **(B,C)** Distal femurs were embedded with paraffin and sliced up, then TRAP staining were performed. The osteoclast number/bone surface (N.Oc/BS, N/mm) was quantified. Data are represented as mean ± SD. *n* = 10 and ^∗^*P* < 0.05. **(D)** The serum level of uPAR was detected by ELISA. Data are presented as mean ± SD. *n* = 10 and ^∗^*P* < 0.05.

We imitated postmenopause bone loss in women by using ovariectomized mice model to evaluate the anti-osteoporosis function of ulinastatin. 6 weeks after the operation, ulinastatin showed no obvious impact on body weight which was recorded every week. In the meanwhile, the groups of OVX and OVX + ulinastatin showed observable atrophy and lessened wet weight of the uterus when compared with SHAM group, which illustrated the success of ovariectomy. In the first place, micro-computed tomography (μ-CT) was utilized to analyze the trabecular bone of distal femoral metaphysis of each mice group, from **Figure 1** we could conclude that bone volume/tissue volume (BV/TV), trabecular thickness (Tb.Th), and trabecular number (Tb.N) in the OVX group notably decreased but increased in trabecular separation (Tb.Sp) when contrasted with the SHAM group, BV/TV, Tb.Th, and Tb.N increased and Tb.Sp decreased in the OVX + ulinastatin group when compared with the OVX group. As it can be seen in the histomorphometric parameters above, ulinastatin possesses the ability to dramatically attenuate ovariectomy-induced trabecular bone loss *in vivo*. In order to reconfirm these results, we performed Hematoxylin and eosin (H&E) staining of the distal femoral metaphysis sections which have been decalcified. It was clearly demonstrated that the trabecular bone in the OVX group was fewer and thinner both distally and proximally to the growth plate than the SHAM group. At the meantime, there was a markedly increase in the OVX + ulinastatin group including bone density, trabecular bone density and thickness when contrasted with OVX group (**Figure 2A**). Afterward, trap staining was performed to investigate whether ulinastatin inhibits bone loss through suppression of osteoclastogenesis *in vivo*. The size and quantity of osteoclasts in OVX group signally elevated when contrasted with those of SHAM group. But when treated with ulinastatin, the size and quantity of osteoclasts in OVX + ulinastatin group decreased dramatically contrasted with OVX group (**Figure 2B**). Histomorphometric parameters analysis verified that the number of osteoclasts per bone surface (N.Oc/BS) elevated in OVX group when contrasted with SHAM group and significantly decreased in OVX + ulinastatin group when contrasted with OVX group (**Figure 2C**).

Furthermore, the serum level of uPAR in OVX group dramatically increased when contrasted with SHAM group, and the uPAR serum level markedly decreased in OVX + ulinastatin group contrasted with OVX group (**Figure 2D**).

Therefore, we could draw a conclusion that ulinastatin can attenuate bone loss induced by OVX through inhibiting osteoclastogenesis, and this process may be highly related with the expression of uPAR.

### Ulinastatin Suppresses RANKL-Induced Osteoclast Formation *in vitro*

**FIGURE 3:**
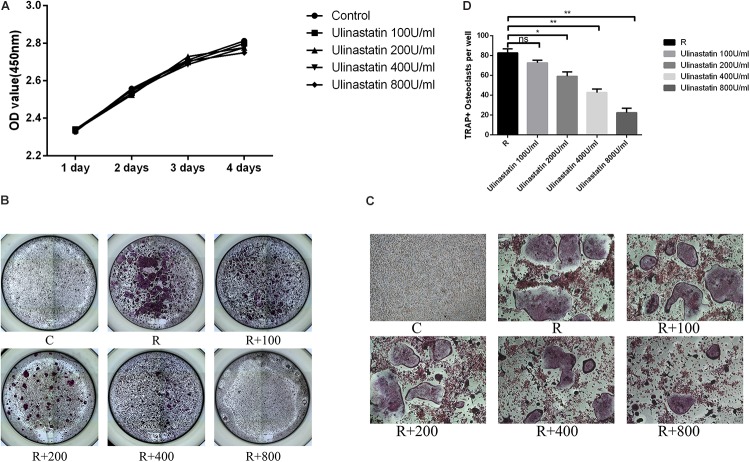
Ulinastatin inhibits RANKL-induced osteoclastogenesis. C represents control group, R represents RANKL group, R + 100 ∼ 800 represents RANKL + 100 units/mL ulinastatin ∼ RANKL + 800 units/mL ulinastatin group. **(A)** Ulinastatin barely has influence on survival and proliferation of BMMs. BMMs (1 × 10^4^ cells/well) were cultured with M-CSF (30 ng/mL) and five different concentrations of ulinastatin (0, 100, 200, 400 and 800 units/mL) for 4 days. Data are presented as mean ± *SD* of three independent experiments. **(B,C)** Ulinastatin prevents the formation of osteoclasts in a dose-dependent manner. BMMs (1.0 × 10^4^ cells/well) were cultured with M-CSF (30 ng/mL), RANKL (100 ng/mL) and four different concentrations of ulinastatin (100, 200, 300 and 400 units/mL) for 5 days. The cells were stained for TRAP assay. **(D)** TRAP-positive multinucleated osteoclasts (≥3 nuclei) were quantified. Data are presented as means ±*SD* of three independent experiments. ^∗^*P* < 0.05 and ^∗∗^*P* < 0.01.

As shown in the result of CCK8 (**Figure 3A**), ulinastatin (ranged from 100 to 800 units/mL) had no detectable influence on proliferation and viability of BMMs. BMMs were treated with four concentrations (100,200,400,800 units/mL) of ulinastatin daily in the presence of M-CSF (30 ng/mL) and RANKL (50 ng/mL) for 5 days. As we can see in **Figures 3B–D** that osteoclast formation can be suppressed by ulinastatin in a dose-dependent manner. When treated by ulinastatin with the concentration of 800 units/mL, the quantity and size of visible TRAP-positive multinucleated cells decreased the most compared with any other group.

### Ulinastatin Inhibits Osteoclast Function

**FIGURE 4:**
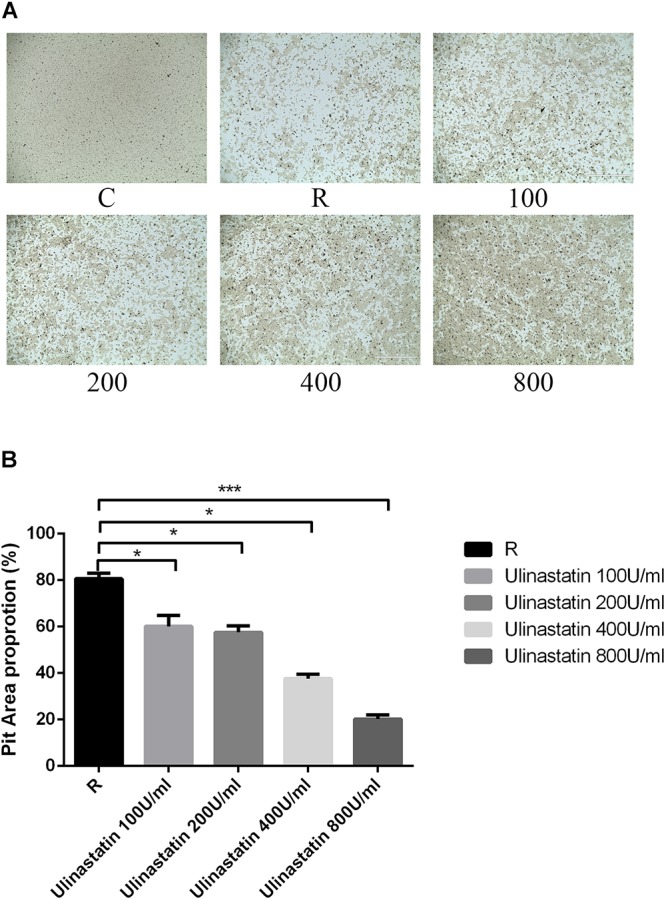
Ulinastatin prevents bone resorption function of osteoclasts. C represents control group, R represents RANKL group, 100 ∼ 800 represents RANKL + 100 units/mL ulinastatin ∼ RANKL + 800 units/mL ulinastatin group. **(A,B)** BMMs (1 × 10^4^ cells/well) were seeded onto a Corning Osteo Assay Surface and cultured with M-CSF (30 ng/mL) and RANKL (100 ng/mL) for 4 days and then treated with four different concentrations of ulinastatin (100, 200, 300, and 400 units/mL) for another 4 days. Then the images were acquired and pit area was quantified. Data are presented as means ± *SD* of three independent experiments; ^∗^*P* < 0.05; ^∗∗^*P* < 0.01; and ^∗∗∗^*P* < 0.001 versus RANKL group.

We preformed pit formation assays, BMMs were seeded onto a bone slice and cultured with M-CSF(30 ng/mL) and RANKL(100 ng/mL) for 5 days (formation of mature osteoclasts), then different concentration of ulinastatin was applied, 4 days later, all cells were taken away and the resorption pits were measured. As we can see in **Figure 4**, ulinastatin dramatically suppressed the bone resorption function of osteoclasts. It demonstrated that ulinastatin inhibits the function of osteoclasts.

### Ulinastatin Suppresses the Expression of Osteoclast-Related Genes

**FIGURE 5:**
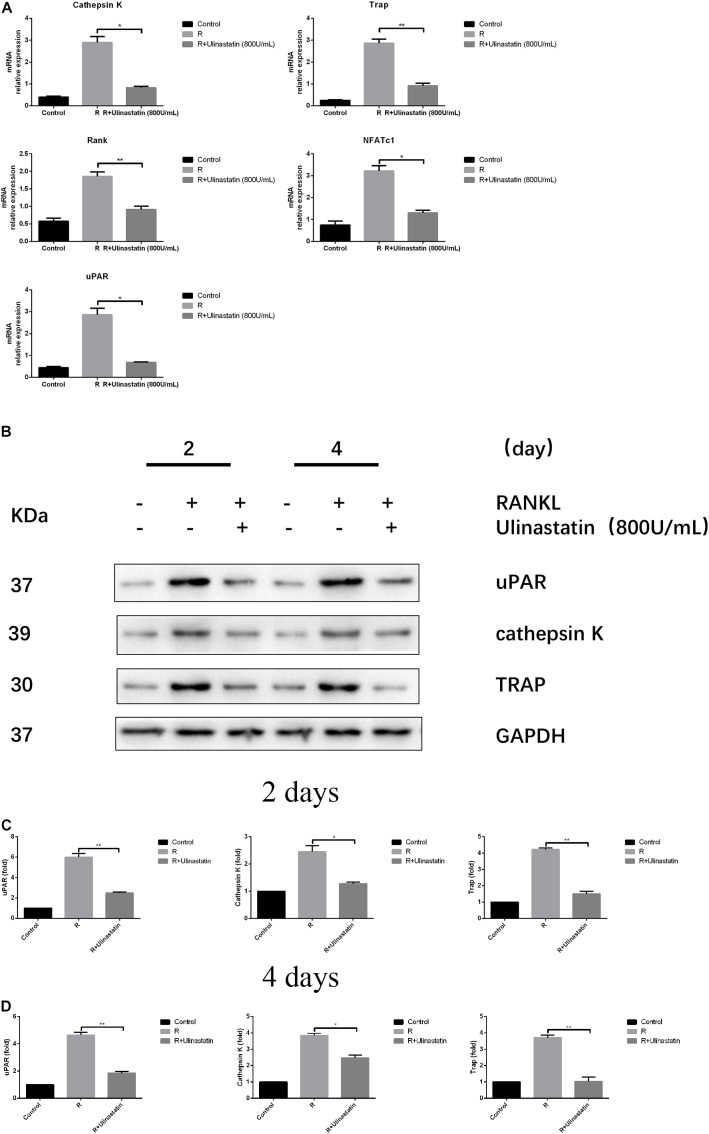
Ulinastatin reduces the expression of uPAR, NFATc1 and osteoclast marker genes induced by RANKL. C represents control group, R represents RANKL group, R + ulinastatin represents RANKL + 800 units/mL ulinastatin group. **(A)** Ulinastatin reduces RANKL-induced mRNA expression of cathepsin K, Trap, Rank, NFATc1, and uPAR. BMMs were cultured with M-CSF (30 ng/mL) and RANKL (100 ng/mL), and treated with or without ulinastatin (800 units/mL) for 4 days. mRNA expression was detected by qRT-PCR. The qRT-PCR experiments have been repeated with different RNA preparations for 3 times independently. Data are represented as mean ± *SD*. ^∗^*P* < 0.05 and ^∗∗^*P* < 0.01. **(B,C,D)** Ulinastatin reduces RANKL-induced protein expression of uPAR, cathepsin K and Trap. BMMs were cultured with M-CSF (30 ng/mL) and RANKL (100 ng/mL), and treated with or without ulinastatin (800 units/mL) for 2 or 4 days. Protein expression levels of uPAR, cathepsin K and Trap were examined by western blotting at the indicated times. The amount of loaded protein was 25 μg. The experiment was performed three times independently and GAPDH was used as a loading control. ^∗^*P* < 0.05, ^∗∗^*P* < 0.01 versus RANKL group.

Bone contains type I collagen, and cathepsin K is a key enzyme in degradation of type I collagen (Konttinen et al., 2001). TRAP is one of the key secreting enzymes of osteoclasts. We detected the expression of cathepsin K and TRAP of BMM treated or not treated with ulinastatin when RANKL was present. The results in **Figures 5B–D** revealed that ulinastatin suppressed the protein expression of cathepsin K and TRAP induced by RANKL. A major transcription factor named NFATc1 (Takayanagi et al., 2002), which is in part managed by its pivotal upstream activator c-Fos during osteoclastogenesis (Matsuo et al., 2004), regulates the differentiation and function of osteoclasts. We tested whether ulinastatin could inhibit the mRNA expression of RANK—the receptor of RANKL, uPAR, and NFATc1-downstream genes including cathepsin K and TRAP (Asagiri and Takayanagi, 2007). As shown in **Figure 5A**, ulinastatin markedly suppressed mRNA expression of cathepsin K, TRAP, NFATc1, RANK, and uPAR.

### uPAR Highly Expresses During Osteoclastogenesis

**FIGURE 6:**
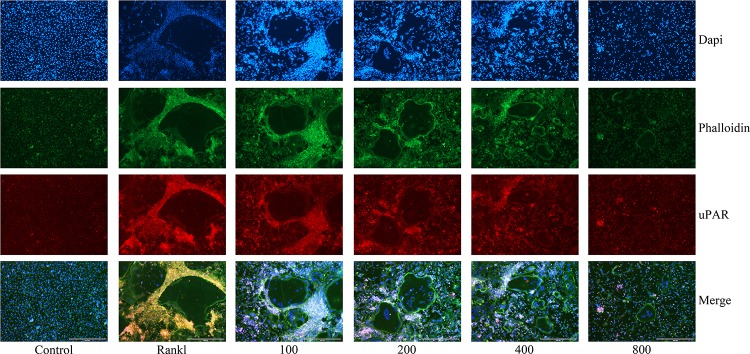
Ulinastatin decreased actin ring formations and suppressed uPAR expression. C represents control group, R represents RANKL group, 100 ∼ 800 represents RANKL + 100 units/mL ulinastatin ∼ RANKL + 800 units/mL ulinastatin group. BMMs (1 × 10^4^ cells/well) were cultured with M-CSF (30 ng/mL) and RANKL (100 ng/mL), treated with four different concentrations of ulinastatin (100, 200, 400 and 800 units/mL) for 4 days. Then the cells were stained for actin ring assay and Immunofluorescence assay. Images were obtained by fluorescence microscopy. The experiment was performed three times independently.

As we have investigated, the protein expression of uPAR increased when BMMs were induced to differentiate into osteoclasts by RANKL, but dramatically decreased when ulinastatin was applied (**Figures 5B–D**). Similarly, from **Figure 6** we can see in the result of immunofluorescence staining that uPAR expression of BMMs increased when treated with RANKL and gradually decreased when treated with increasing concentration of ulinastatin. It can be seen from the results that uPAR is highly expressed during the formation of osteoclasts, and therefore uPAR may play an extremely important role in the process of osteoclastogenesis.

Actin ring, which affects osteoclast adhesion and bone resorption, plays a crucial role in osteoclast function (Ke et al., 2012). To further investigate the impact ulinastatin had on the formation of actin ring, we performed actin ring formation assays. According to the result of phalloidin staining (**Figure 6**), it is obvious that with the elevated concentration of ulinastatin, its inhibiting effect on actin ring formation gradually enhanced.

### Knockdown of uPAR Inhibits Osteoclast Formation and Function

**FIGURE 7:**
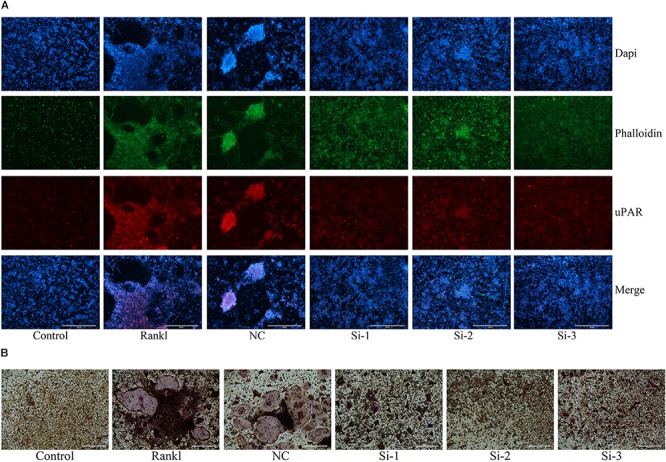
Knockdown of uPAR decreased RANKL-induced osteoclast and actin ring formation. NC represents siRNA negative control, Si-1, Si-2, and Si-3 represent siRNAs that silence three different fragment of uPAR. BMMs were seeded in 96-well plates and transfected with three siRNAs and NC siRNA, respectively, and cultured with M-CSF (30 ng/mL) and RANKL (50 ng/mL) for 5 days. **(A)** Immunofluorescence and **(B)** TRAP staining was performed then. The experiment was performed three times independently.

In order to reconfirm the relationship between ulinastatin, uPAR and osteoclastogenesis, we explored the role which uPAR plays in osteoclast formation. siRNAs that silenced three different fragments of uPAR were transfected into BMMs, and then the BMMs were cultured with 50 ng/mL RANKL and 30 ng/mL M-CSF for 5 days. As shown in **Figure 7A**, after blocked by siRNA, the expression of uPAR and the formation of actin ring dramatically decreased. **Figure 7B** shows that three different uPAR siRNAs have the ability to completely prevent the formation of osteoclast. Combine all the results above, we can conclude that the expression of uPAR largely affects osteoclastogenesis, and this process can be regulated by ulinastatin.

### Ulinastatin Inhibits RANKL-Induced MAPKs and NF-κB Activation

**FIGURE 8:**
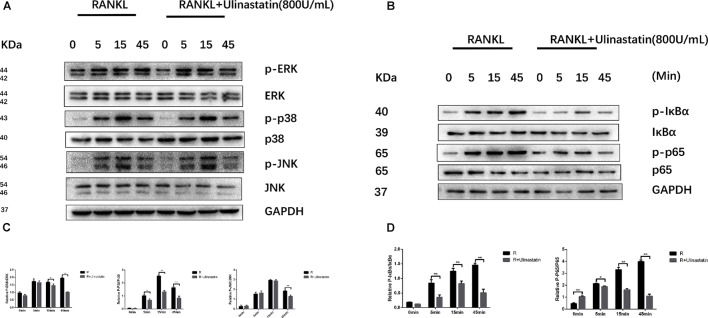
Ulinastatin suppressed RANKL-induced activation of MAPKs and NF-κB. **(A,C)** Ulinastatin represses RANKL-induced phosphorylation of ERK, p38, and JNK. BMMs were starved with α-MEM basic medium free from FBS for 12 h, and pretreated with or without ulinastatin (800 units/mL) for 1 h. Then BMMs were treated with or without RANKL (100 ng/mL) and Ulinastatin (800 units/mL) for the indicated times. The total proteins were extracted and examined by western blotting. **(B,D)** Ulinstatin represses RANKL-induced phosphorylation of IκBα and p-65. BMMs were treated as described above, and the total proteins were extracted and examined by western blotting. The experiment was performed three times independently and the total protein was used as a loading control. ^∗^*P* < 0.05 and ^∗∗^*P* < 0.01 versus RANKL group.

The process of osteoclastogenesis needs the activation of MAPKs and NF-κB signaling pathways (Takayanagi et al., 2002; Bruzzaniti and Baron, 2006; Takayanagi, 2007a). In order to confirm whether the inhibition function of ulinastatin on osteoclast differentiation and maturity is through these two pathways, further research was carried out. According to our western blot results, ulinastatin inhibited degradation of IκBα and phosphorylation of IκBα and NF-κB p65 induced by RANKL (**Figures 8B,D**). As to MAPKs, ulinastatin can also inhibit the phosphorylation of JNK, ERK and p38 (**Figures 8A,C**). Taken together, ulinastatin has the ability to inhibit the activation of NF-κB and MAPKs.

## Discussion

According to our present study, ulinastatin could effectually inhibit osteoclastogenesis both *in vitro* and *in vivo*, and effectively suppress bone loss. We have already demonstrated that ulinastatin attenuated bone loss from different aspects. Ulinastatin could restrain the formation of osteoclast and actin ring. Besides, the function of osteoclast was dramatically suppressed in the presence of ulinastatin. As for the molecular level, ulinastatin possessed the ability to suppress multiple downstream pathways of RANK, including MAPKs, NFATc1, NF-κB, and AP-1. Moreover, ulinastatin was testified to reduce the serum level of uPAR, and knockdown of uPAR could greatly repress the formation of osteoclast.

The formation and function of osteoclast is regulated by one of the most crucial downstream pathway–NF-κB (Takayanagi, 2007a). As we already know, activation of NF-κB leads to the increase of proinflammatory cytokines which can induce osteoclastogenesis (Zhou et al., 2006). Thus, NF-κB signaling pathway could be the convergent point between inflammatory and osteoclastogenesis. Blocking of NF-κB inhibits osteoclast formation and stems inflammation bone destruction *in vivo* (Jimi et al., 2004). As confirmed by inhibition of phosphorylation of P65 and IκBα, we believe that NF-κB mediates the inhibition effect of ulinastatin on osteoclastogenesis. Upstream signaling pathway of NF-κB–MAPKs, which can be stimulated by RANKL, is related to osteoclastogenesis. We explored that ulinastatin markedly suppressed phosphorylation of ERK, P38, and JNK initiated by RANKL. AP-1 consists of c-Fos and c-Jun and the activation of AP-1 launched by MAPKs facilitates further autoamplification of NFATc1 (Gohda et al., 2005), which in turn increases the expression of its downstream osteoclast-related genes. Additionally, our results revealed that ulinastatin evidently inhibited protein expression of cathepsin K and TRAP induced by RANKL, and receded the activation of osteoclast-related marker genes, including NFATc1, TRAP, cathepsin K, and RANK, which are involved in bone resorption and bone remodeling (Asagiri and Takayanagi, 2007). Taken all together, we can conclude that multiple signaling pathways mediate inhibition effect of ulinastatin on osteoclast formation and function.

uPAR-directed signaling can occur via uPA-uPAR binding or be uPA-independent manner. Although uPAR lacks transmembrane and intracellular domains, uPAR can be considered to be one of the transmembrane receptors that constructs an active complex with integrins and plays a vital role in mediating integrin-mediated downstream signaling related to changes in structure of cytoskeleton, cell adhesion, migration, and metastasis (Mondino et al., 1999; Smith and Marshall, 2010). Signaling through uPAR can activate the Ras–MAPK pathway, the Tyr kinases focal adhesion kinase (FAK), Rho family small GTPase and phosphatidylinositide 3-kinase (PI3K)–Akt pathway (Smith and Marshall, 2010). For example, uPAR–β1 integrin communications are frequently accompanied with the activation of FAK and ERK, and uPAR–β3 integrin interactions are associated with the activation of Rac (Aguirre, 2002; Liu et al., 2002; Wei et al., 2008). Already published research showed that uPAR potentiates the RANK/RANKL-induced osteoclastogenesis of mouse monocyte/macrophage linage cells in a PI3K/Akt-dependent manner (Kalbasi et al., 2015). In our study, we have confirmed that uPAR expression was elevated during osteoclast formation induced by RANKL. In addition, we can also see that uPAR mainly overexpresses around osteoclasts during osteoclast formation. According to our observation, osteoclastogenesis *in vitro* involved the migration of BMMs. Therefore, we speculated that during osteoclastogenesis, uPAR acted a crucial part in regulating the migration of BMMs and interdicting this process could markedly inhibit osteoclastogenesis as shown in our research. Known as a valid anti-inflammatory drug, ulinastatin could recede uPAR expression through MAPKs and NF-κB signaling pathways according to our study. All these outcomes showed that uPAR could be a pivotal factor mediated by ulinastatin during its inhibiting effect on osteoclastogenesis.

What cannot be ignored is that important limitations exist in our present study, and the limitations can also represent the future directions we should explore. As we already know, bone is constantly undergoing remodeling including bone formation and bone resorption caused by osteoclastogenesis and osteoblastogenesis (Boyle et al., 2003). Our study clarified that ulinastatin inhibited OVX-induced osteoclastogenesis and bone loss, however, whether ulinastatin facilitates osteoblastogenesis still needs to be investigated. Besides, as a crucial mediate factor of ulinastatin, how uPAR concretely functions in osteoclastogenesis needs to be further researched.

As a commonly used clinical drug, ulinastatin is mainly used for acute pancreatitis and chronic recurrent pancreatitis (Ohwada et al., 1997; Wang et al., 2016). Recent studies show that ulinastatin is also beneficial in treatment of cerebral ischemia/reperfusion injury (Cao et al., 2011; Liu M. et al., 2017), early cardiopulmonary resuscitation, lung injury induced by hip fracture and pulmonary fibrosis as well as others (Liu Q. et al., 2017). Our research confirmed that ulinastatin has an effective inhibitory impact on osteoclastogenesis both *in vivo* and *in vitro* and OVX-induced bone loss. To sum up, our findings suggests that ulinastatin may serve as a potential therapeutic strategy for diseases related to osteoclasts such as postmenopausal osteoporosis, rheumatoid arthritis, and Paget’s disease (Takayanagi, 2007a; Novack and Teitelbaum, 2008). Considering the inconvenience brought by osteoporosis to people’s daily life as well as the disadvantages of existing osteoporosis drugs, it is important and urgent to ascertain whether ulinastatin can be chosen as an effective alternative therapeutics for osteoclast-related disease.

## Materials and Methods

### Reagents and Antibodies

Ulinastatin was purchased from Techpool (Guangzhou, China), and dissolved in normal saline and serum-free α-MEM basic medium for using *in vivo* and *in vitro*, respectively. Recombinant soluble mouse RANKL and macrophage-colony stimulating factor (M-CSF) were obtained from Peprotech (Rocky Hill, CT, United States). Rabbit antibodies against p38(D13E1, #8690), p-p38(D3F9, #4511), ERK(137F5, #4695), p-ERK(D13.14.4E, #4370), JNK (#9252), p-JNK(81E11, #4668), p65(D14E12, #8242), p-p65(93H1, #3033), IκBα(L35A5, #4814), and p-IκBα(14D4, #2859) were acquired from Cell Signaling Technology (Boston, MA, United States). Mouse antibody against uPAR (ab103791) was obtained from Abcam (Cambridge, MA, United States). Mouse antibody against GAPDH (A00227-1, P04406) was obtained from Boster (Wuhan, China).

### Animals

Three months old C57/BL6 female mice were purchased form the Experimental Animal Center of Tongji Hospital, Huazhong University of Science and Technology (Wuhan, China), and kept in the animal care facility of Tongji Hospital. The living environment of mice was maintained at 25°C with 12:12 light/dark cycle, and mice were fed with normal chow and water.

Mice were separated into three groups (*n* = 10 mice/group) at random: bilateral ovariectomized mice intraperitoneal injected with ulinastatin (1 × 10^5^ units/kg per day) (OVX + ulinastatin), bilateral ovariectomized mice intraperitoneal injected with isopyknic DMSO (OVX), sham-operated mice intraperitoneal injected with isopyknic DMSO (SHAM). The injection dose of ulinstatin refers to previous studies and the dose is similar to therapeutic dose (Shen et al., 2014; Li et al., 2015). Ulinstatin solution and DMSO were administered by intraperitoneal injection in the mice seven times a week for 6 weeks. The injection of ulinastatin and DMSO started on the first day after surgery. The weights of all mice were recorded every week. Six weeks after drug administration the mice were sacrificed and the orbital blood, uteruses, femurs, and tibias were collected for the following experiments. All experimentations on animals have been reviewed and approved by the Ethics Committee on Animal Experimentation of Tongji Medical college, Huazhong University of Science and Technology (Wuhan, China), and experiments involving human participants have also been approved by the same Ethics Committee.

### Micro-Computed Tomography (μ-CT)

After removing soft tissue, we used micro-computed tomography system (μ-CT50 Scanco Medical, Basserdorf, Switzerland) to scan the distal femoral cancellous bone. Image procurement was performed at 98 μA and 100 kV with a resolution of 10.5 μm. Three dimensional reconstruction and then the evaluation of BV/TV, Tb.Th, Tb.N, and Tb.Sp were all performed in μ-CT system.

### Histomorphometric Analysis

The femur samples separated from the mice for histomorphometric analysis were dipped in 4% paraformaldehyde for 48 h for fixation and then dipped in 10% EDTA solution for 2 weeks for decalcification. When decalcification was finished, the bones were embedded in paraffin wax and sliced up. Half of the sections were used for H&E staining for observation of trabecular structure. The other half of the sections were used for TRAP staining (Sigma-Aldrich, MO, United States) so that osteoclasts’ number in the region could be counted as mentioned earlier (Lee et al., 2006; Guan et al., 2015).

### Serum Biochemistry

Before sacrifice, orbital blood of mice was drawn, and sera were extracted immediately. The serum level of uPAR was evaluated by mouse uPAR ELISA kit (Boster, Wuhan, China).

### Cell Cultures

As described previously, BMMs were flushed out from the tibias and femurs bone marrow cavities of 4–6 weeks old mice with α-MEM medium (Koga et al., 2004; Zhang et al., 2015). The α-MEM medium contained FBS (10%), M-CSF (30 ng/mL), streptomycin (100 μg/mL), and penicillin (100 U/mL). BMMs were cultured in the medium, 24 h later, cells, which did not adhere to the bottom of the bottle, were collected, further culture and used as BMMs.

### Cell Counting Kit-8 Assay

In order to estimate cell viability and proliferation, we performed cell counting kit-8 assay (CCK8 assay, Boster). We seeded BMMs in 96-well plates (1 × 10^4^ cells/well). 24 h later, different concentrations of ulinastatin (100, 200, 400, and 800 U/mL) with M-CSF (30 ng/mL) were applied daily for 4 days. After 1, 2, 3 and 4 days, the original culture medium was discarded and fresh medium containing 10% CCK-8 was added into the plates. BMMs were incubated at 37°C for 1 h, then we used ELX800 absorbance microplate reader (Bio-Tec, VI, United States) to measure the absorbance at 450 nm wavelength.

### Actin Ring Formation Assays and DAPI Staining

BMMs were seeded into a 96-well plate (1 × 10^4^ cells/well), 24 h later they were cultured with RANKL (100 ng/mL) and different concentrations of ulinastatin for 5 days. Next, culture medium was replaced with immune staining fix solution (Beyotime, Shanghai, China) for 10 min, in order to permeabilize the cells. Then they were incubated with immune staining wash buffer (Beyotime, Shanghai, China) for 5 min and incubated at 25°C with phalloidin (Sigma-Aldrich, MO, United States) for another 30 min so that F-actin will visualize. After that, cells were washed by using phosphate buffer saline for four times and then DAPI (Boster, Wuhan, China) was applied for 5 min. Images were acquired by using fluorescence microscope.

### Immunofluorescence Staining

BMMs were seeded into a 96-well plate (1 × 10^4^ cells/well), 24 h later RANKL (100 ng/mL) and different concentrations of ulinastatin were, respectively, applied. 5 days later, culture medium was replaced with immune staining fix solution (Beyotime, Shanghai, China) for 10 min, in order to permeabilize the cells. Then the cells were incubated with 1% Triton for 30 min followed by goat serum for 20 min at 37°C. After that the cells were incubation with antibody against uPAR (Proteintech, Wuhan, China) for overnight at 4°C followed by incubation with cy3 anti-rabbit fluorescent secondary antibody (Proteintech, Wuhan, China) at 37°C for 1 h. Images were obtained by using fluorescence microscope.

### *In vitro* Osteoclastogenesis Assay

BMMs were planted into 96-well plates (1 × 10^4^ cells/well) with the presence of M-CSF (30 ng/mL) and RANKL (100 ng/mL), treated with or without different concentrations of ulinastatin for 5 days and the culture medium was replaced every single day. Then we performed TRAP staining, trap-positive multinucleated cells (≥3 nuclei) were identified as osteoclasts (Asagiri and Takayanagi, 2007). Images were acquired by using EVOS FL auto cell image system (Life Technologies, United Kingdom).

### Pit Formation Assays

BMMs were seeded (20,000 cells/well) into a Corning Osteo Assay Surface plate (Corning Incorporated Life Science, NY, United States), cultured with RANKL (100 ng/mL) and M-CSF (30 ng/mL) for 5 days (until the quantity and size of osteoclasts were large enough), and then different concentrations of ulinastatin were applied or not. Another 4 days later, the plate was washed by using 5% sodium hypochlorite and PBS for 5 and 10 min, respectively (Guan et al., 2015). The images of bone resorption pits were acquired through microscopy, and then measured and quantified.

### RNA Extraction and Quantitative Real-Time Reverse Transcription-PCR

Quantitative real-time reverse transcription PCR was carried out as described before (Zhang et al., 2015). BMMs were planted into a 6-well plate (1 × 10^5^ cells/well), cultured with the medium containing RANKL (100 ng/mL) and M-CSF (30 ng/mL), treated with or without ulinastatin (800 U/mL). The extraction of total RNA was conducted through E.Z.N.A Total RNA Kit I (Omega Bio-Tek, Norcross, GA, United States). Reverse transcription was conducted by using Rever Tra Ace qPCR RT Kit (Toyobo, Osaka, Japan), qRT-PCR was conducted by using SYBR qPCR Mix (Toyobo, Osaka, Japan) on Bio Rad Q5 instrument (Bio-Rad Laboratories, CA, United States), and the target genes’ expression was standardized to the reference gene GAPDH. All procedures were conducted following manufacturer’s protocol. Primers used for qRT-PCR (F represents forward; R represents reverse) : GAPDH (F) 5′-CTCCCACTCTTCCACCTTCG-3′, (R) 5′-TTGCTGTAGCCGTATTCATT-3′; TRAP (F) 5′-TACCTGTGTGGACATGACC-3′, (R) 5′-CAGATCCATAGTGAAACCGC-3′; Cathepsin K (F) 5′-TGTATAACGCCACGGCAAA-3′, (R) 5′-GGTTCACATTATCACGGTCACA-3′; NFATc1 (F) 5′-CAACGCCCTGACCACCGATAG-3′, (R) 5′-GGGAAGTCAGAAGTGGGTGGA-3′; RANK (F) 5′-CAGGAGAGGCATTATGAGCA-3′, (R) 5′-GGTACTTTCCTGGTTCGCAT-3′; uPAR (F) 5′-AACTCAGCCTCATTGCCTCT-3′, (R) 5′-TCCTCAAAGATGGAGCAGGG-3′.

### Western Blot Analysis

Western blot analysis was conducted as described before (Dong et al., 2016, 2017). The cells, seeded in 6-well plates and have been treated or not, were lysed with RIPA lysis solution (Boster) containing broad spectrum phosphatase inhibitors and PMSF (Boster). Total concentration of proteins was measured by BCA assays (Boster, Wuhan, China). The electrophoresis of proteins was conducted in 10% SDS-polyacrylamide gel and then the proteins were transferred to the PVDF membranes (Millipore, MA, United States). 5% bovine serum albumin was used for blocking of the membranes, then the membranes were incubated with respective antibodies overnight. After washed, the membranes were incubated at 25°C for 1 h with horseradish peroxidase-conjugated secondary antibodies (Boster). Subsequently, enhanced chemiluminescence (Boster) was used to visualize the proteins, and images were acquired through ChemiDoc^TM^ XRS+ System with Image Lab^TM^ Software (Bio-Rad Laboratories, CA, United States).

### Small Interfering RNA (siRNA) Assays

BMMs were seeded in a 96-well plate (10,000 cells/well), and then were transfected with 20 μM siRNAs by using siRNA Transfection Reagent (Ribbio, Guangzhou, China)with 30 ng/mL M-CSF according to the manufacturer’s protocol. Four hours later, the incubating siRNA mix was replaced with α-MEM culture medium containing M-CSF (30 ng/mL) and RANKL (50 ng/mL). After the BMMs were cultured for 5 days, TRAP staining was performed. siRNAs used for siRNA assays : si-1 GGACCATGAGTTACCGCAT; si-2 CCATAGCAACCAGACCTTT; si-3 GCTGGGAAACCGGAGTTAT.

### Statistical Analysis

All experiments were independently repeated more than three times, and the results were presented as means ±*SD*. We performed Student’s *t*-test for the compare between two groups and one-way ANOVA for more than two groups. ^∗^*P* < 0.05 and ^∗∗^*P* < 0.01 were considered as statistical significant difference.

## Author Contributions

HK, F-jG, J-mH, and R-yR conceived and devised the study. J-mH, R-yR, and YB performed the experiments. WX, G-xZ, JS, and J-cG analyzed the data. J-mH, R-yR, and YB wrote the paper. LL and YT revised the manuscript. HK obtained the funding and supervised the whole project. All authors have contributed to the final version and approved the publication of the final manuscript.

## Conflict of Interest Statement

The authors declare that the research was conducted in the absence of any commercial or financial relationships that could be construed as a potential conflict of interest.
