# Corrigendum: Ulinastatin Inhibits Osteoclastogenesis and Suppresses Ovariectomy-Induced Bone Loss by Downregulating uPAR

**DOI:** 10.3389/fphar.2018.01128

**Published:** 2018-10-09

**Authors:** Jun-ming Huang, Ran-yue Ren, Yuan Bao, Jia-chao Guo, Wei Xiang, Xing-zhi Jing, Jia Shi, Guo-xiang Zhang, Long Li, Yong Tian, Hao Kang, Feng-jin Guo

**Affiliations:** Department of Orthopedics, Tongji Hospital, Tongji Medical College, Huazhong University of Science and Technology, Wuhan, China

**Keywords:** ulinastatin, uPAR, osteoclast, MAPKs, NF-kB, RANKL, osteoporosis

In the original article, there was a mistake in Figure 1 as published. The images used for the group of OVX+U were from a different study. The corrected Figure 1 appears below. The authors apologize for this error and state that this does not change the scientific conclusions of the article in any way.

**Figure 1:**
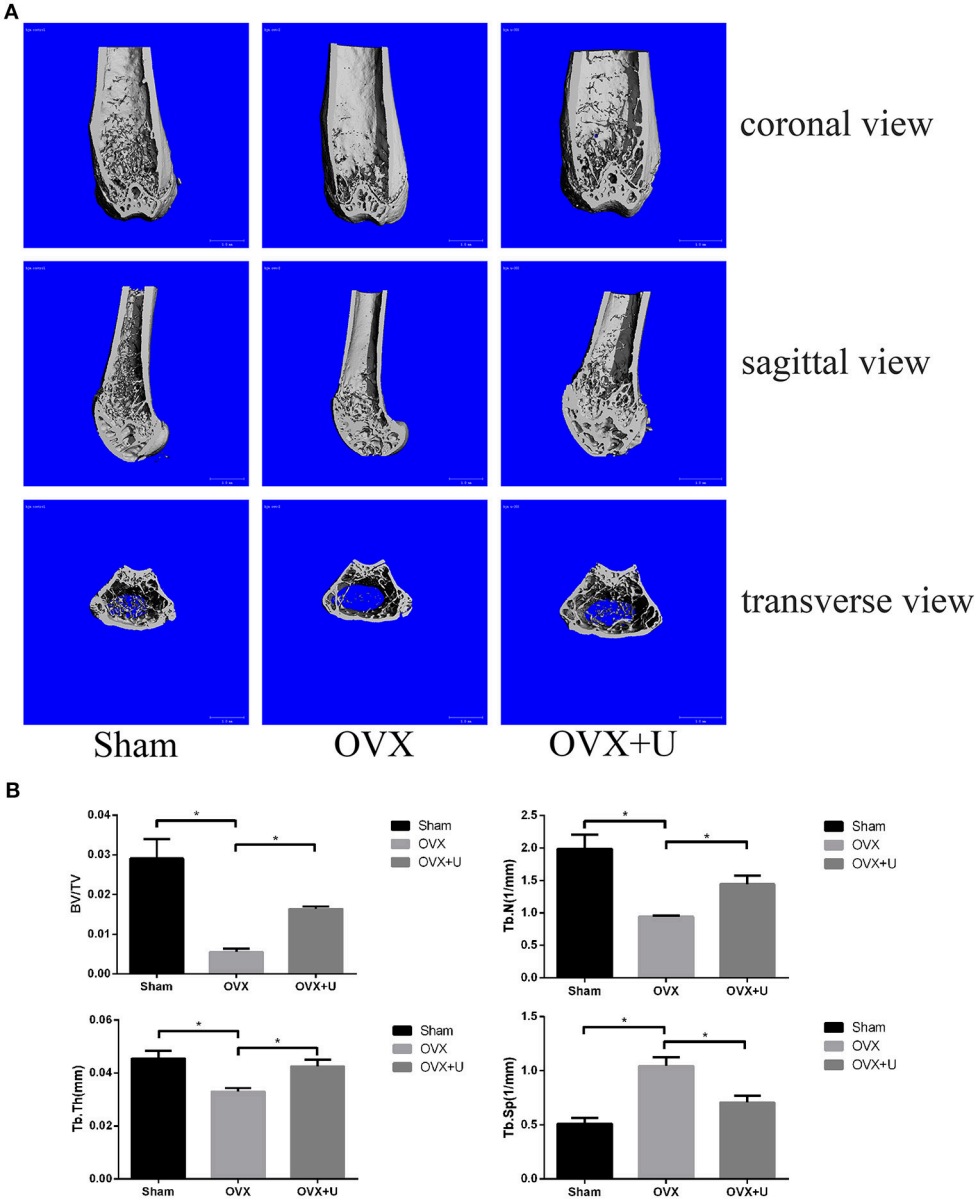
Ulinastatin inhibits bone loss induced by OVX. **(A)** μ-CT images of the trabecular bone of distal femoral metaphysis from the SHAM, OVX, and OVX + ulinastatin groups. **(B)** The trabecular structural parameters of the distal femur: trabecular bone volume/tissue volume (BV/TV), trabecular number (Tb.N), trabecular thickness (Tb.Th), and trabecular separation (Tb.Sp). Data are presented as means ± *SD*. *n* = 10 and ^*^P < 0.05.

The original article has been updated.

## Conflict of interest statement

The authors declare that the research was conducted in the absence of any commercial or financial relationships that could be construed as a potential conflict of interest.

